# Heart rate elevations during early sepsis predict death in fluid-resuscitated rats with fecal peritonitis

**DOI:** 10.1186/s40635-018-0190-5

**Published:** 2018-08-20

**Authors:** Alain Rudiger, Victor Jeger, Mattia Arrigo, Christian A. Schaer, Florian F. Hildenbrand, Margarete Arras, Burkhardt Seifert, Mervyn Singer, Gabriele Schoedon, Donat R. Spahn, Dominique Bettex

**Affiliations:** 1 Institute of Anesthesiology, University and University Hospital Zurich, Raemistrasse 100, 8091 Zurich, Switzerland; 2 Inflammation Research Unit, Division of Internal Medicine, University and University Hospital Zurich, Raemistrasse 100, CH 8091 Zurich, Switzerland; 3 Clinic for Cardiology, University Heart Centre, University and University Hospital Zurich, Raemistrasse 100, CH 8091 Zurich, Switzerland; 4 Department of Surgery, University and University Hospital Zurich, Raemistrasse 100, CH 8091 Zurich, Switzerland; 5 Department of Biostatistics at Epidemiology, Biostatistics and Prevention Institute, University of Zurich, Hirschengraben 84, 8001 Zurich, Switzerland; 6 Bloomsbury Institute of Intensive Care Medicine, Division of Medicine, University College London, Gower Street, London, WC1E 6BT UK

**Keywords:** Sepsis, Animal model, Fecal peritonitis, Telemetry, Electrocardiogram, Heart rate, Outcome

## Abstract

**Background:**

In sepsis, early outcome prediction would allow investigation of both adaptive mechanisms underlying survival and maladaptive mechanisms resulting in death. The aim of this study was to test whether early changes in heart rate monitored by telemetry could predict outcome in a long-term rat model of fecal peritonitis.

**Methods:**

Male Wistar rats (*n* = 24) were instrumented with a central venous line for administration of fluids, antibiotics and analgesics. A telemetry transmitter continuously collected electrocardiogram signals. Sepsis was induced by intraperitoneal injection of fecal slurry, and the animals were observed for 48 h. Additional animals underwent arterial cannulation at baseline (*n* = 9), 4 h (*n* = 16), or 24 h (*n* = 6) for physiology and laboratory measurements.

**Results:**

48-h mortality was 33% (8/24), with all deaths occurring between 4 and 22 h. Septic animals were characterized by lethargy, fever, tachycardia, positive blood cultures, and elevated cytokine (IL-1, IL-6, TNF alpha) levels. An increase in heart rate ≥ 50 bpm during the first 4 h of sepsis predicted death with sensitivity and specificity of 88% (*p* = 0.001).

**Conclusions:**

In this long-term rat sepsis model, prognostication could be made early by telemetry-monitored changes in heart rate. This model enables the study of underlying mechanisms and the assessment of any differential effects of novel therapies in predicted survivors or non-survivors.

## Background

Sepsis, the dysregulated host response to infection leading to organ dysfunction, is a common and frequently fatal condition [1], with as many annual deaths as those from acute myocardial infarction [1, 2]. Despite major efforts over the last decade, mortality rates still range between 25 and 50% [1, 3, 4]. Hence, a better understanding of the underlying pathophysiology and new therapeutic concepts are urgently needed to improve outcome. To date, mechanistic studies have been predominantly performed with comparison against non-septic control (sham) animals. However, this approach has the important limitation of not discriminating between adaptive *mechanisms of survival* and maladaptive *mechanisms of death*. Early prediction of outcome in an individual animal would enable such discrimination. It would also greatly facilitate the testing of novel therapies to determine any survival impact in likely non-survivors while focusing on safety and the rate/degree of recovery in likely survivors.

We previously reported that echocardiography-derived stroke volume and heart rate could prognosticate as early as 3–6 h in a 3-day rat model of fecal peritonitis [5]. Echocardiography however requires anesthesia, expensive equipment, and experienced investigators. Heart rate can also be measured in real-time by telemetry, with continuous monitoring of the electrocardiogram (ECG) in awake animals. In addition, ECG telemetry provides information on heart rate variability and documents the precise time of demise. The aim of the current study was to test whether telemetry-monitored changes in heart rate during early sepsis could predict outcome in a long-term (48 h) rat model of fecal peritonitis. For clinical relevance, this model received fluid resuscitation and antibiotics, and analgesia to meet the high requirements of animal welfare.

## Methods

### Animal model

All animal experiments were performed in the animal laboratories of the University Hospital Zurich, Switzerland. All protocols were approved by the Animal Experimentation Committee of the Veterinary Office of the Canton of Zurich, Switzerland. Principles of the 3Rs were implemented [6, 7].

**Fig. 1:**
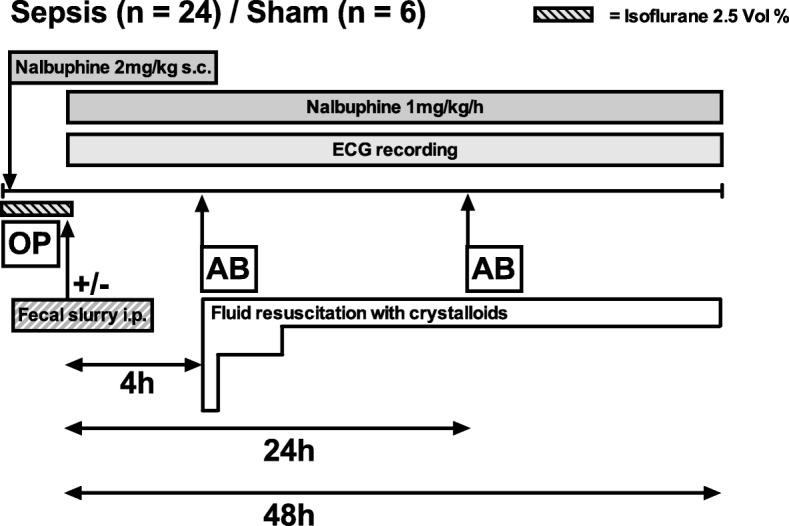
Timeline long-term experiments including heart rate monitoring and mortality. AB antibiotics; OP operation (instrumentation)

**Fig. 2:**
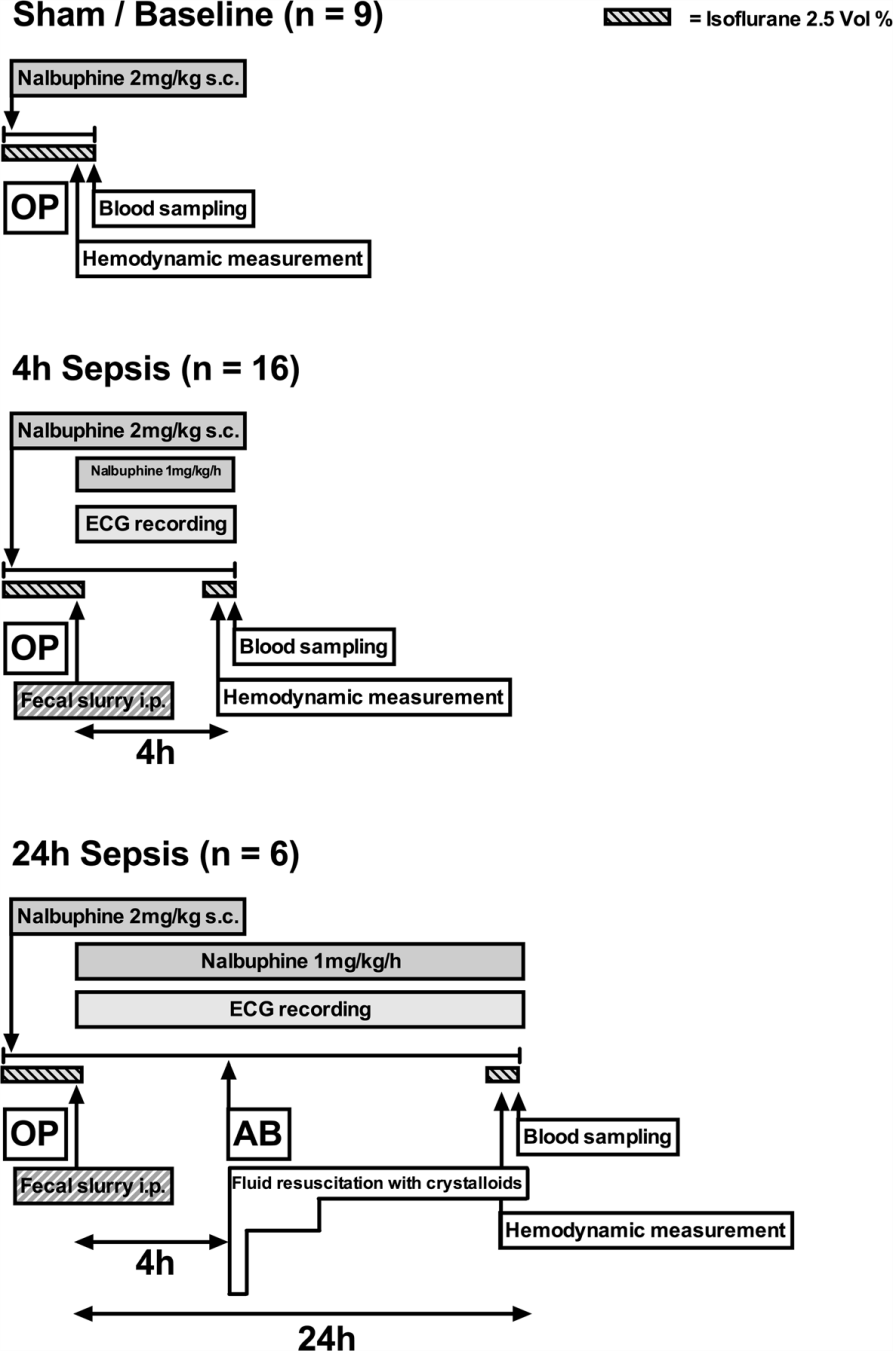
Timeline for short-term experiments including hemodynamic measurements and blood sampling. The 4 h timepoint is prior to fluid resuscitation. AB antibiotics; OP operation (instrumentation)

The current animal model has been adapted from a model of fecal peritonitis, which is established and still being in use at University College London, UK [5, 8, 9]. Figures 1 and 2 summarize the timelines and numbers of animals (n) for each group. A total of 61 animals were included in this study (48 h outcome study: sepsis *n* = 24 and sham *n* = 6; physiology and laboratory measurement study: baseline *n* = 9; 4 h sepsis *n* = 16; 24 h sepsis *n* = 6).

Under general anesthesia (2.5% isoflurane in room air), male Wistar rats were instrumented with a central venous line and a swivel-tether system allowing the rat, after recovery from anesthesia, to have unimpeded movement in its cage. Details on instrumentation have been reported previously [10].

The animals received the opioid analgesic nalbuphine subcutaneously prior to instrumentation (2 mg/kg) and as a continuous infusion thereafter (1 mg/kg/h) [10]. The rationale for the use of nalbuphine has been summarized recently [11].

While still under anesthesia, sepsis was induced by an intraperitoneal injection of 2 mL/kg fecal slurry (25% suspension). Fecal slurry contained feces collected from several animals of the same batch that was suspended in Ringer’s acetate, and then filtered. Therefore, our model has two interventions, namely, surgery (comprising cannulation of the internal jugular vein and subcutaneous implantation of the telemetry device), and injection of fecal slurry into the peritoneal cavity immediately thereafter. Sham animals received fluids and nalbuphine, but did not receive any intraperitoneal injection.

Fluid resuscitation with Ringer’s acetate was commenced 4 h after the septic insult through the central venous line. After a fluid challenge of 20 mL/kg given over 15 min, crystalloids were infused at a rate of 10 mL/kg/h between 4 and 8 h. At 8 h, the infusion rate was reduced to 5 mL/h, and at 24 h halved again to 2.5 mL/h. This fluid resuscitation protocol was adapted from an established animal model [5, 8] by replacing the 1:1 solution of 6% hetastarch and glucose with crystalloids and reducing the overall amount of fluids administered.

Ceftriaxone 30 mg/kg was given intravenously 4 and 24 h after the septic insult [12]. To confirm that our animals developed clinically relevant infection, two additional animals were instrumented as described above, and received fecal slurry but no antibiotics. 6 h after sepsis induction, the blood and peritoneal fluids were sampled for microbiological analyses performed at the Institute for Medical Microbiology, University Zurich.

Baseline values of heart rate were collected 15 min after the end of instrumentation, when the animal had recovered from general anesthesia. Observation time in the long-term experiments was 48 h. Time of death was defined by asystole in the ECG.

For physiology and laboratory measurements, animals were prepared as described above (Fig. 2). At baseline, 4 h (prior to fluid resuscitation) or 24 h, a catheter was placed in the right carotid artery under isoflurane anesthesia, which allowed blood pressure measurements and arterial blood sampling. The arterial line was attached to a pressure transducer (ADInstruments, Oxford, UK), as previously described [10].

Blood gas analysis was performed with an Epoc blood analyzer (Epocal Inc. Ottawa, Ontario, Canada). Plasma cytokines were analyzed with a Bio-Plex Pro Rat cytokine 24-plex assay using the Bio-Plex 200 Suspension Array System (Bio-Rad Laboratories AG, Cressier, Switzerland). B-type natriuretic peptides (BNP) and troponin ITC complex were measured using the Rat BNP and Rat Troponin ITC Complex Assay kits (MesoScale Discovery, Rockville, MD, USA) according to the manufacturers’ instructions.

### Heart rate measurements

Under general anesthesia, a telemetry transmitter (Model RT50B, Millar, Houston, TX, USA) was implanted subcutaneously under the xyphoid process. Two electrodes were placed subcutaneously to either side of the chest, as previously described [10]. No prophylactic antibiotics were given for this procedure.

ECG signals were collected by a SmartPad TR181 (Millar) at a sampling rate of 1 kHz and then transferred via a PowerLab (ADInstruments) to a PC. Heart rate and heart rate variability were analyzed with LabChart V7 (ADInstruments) by a blinded investigator (MA) using published guidelines [13]. Ectopic beats and artifacts were excluded from analysis. R-R intervals were classified as normal (NN) between 100 and 250 ms. A NN difference > 50 ms was defined as the variation threshold. Settings included a FFT size of 1024 and a window according to Hann [14]. We focused our analysis of HRV on SDNN (standard deviation of normal-normal (NN) intervals (=R-R intervals); reflecting cyclic components of sympathetic and parasympathetic activity); total power, the variance of all NN intervals; and the ratio of LF/HF, where LF represents the power of low-frequency range (sympathetic response) and HF the power of high-frequency range (parasympathetic response) [14].

### Statistics

All results are indicated as mean ± standard deviation (SD) with the exception of Figs. 4 and 5 where standard error of the mean (SEM) was used to enhance readability. Assuming a sepsis mortality of 25–50% in the long-term study, a sample size of 24 septic animals was calculated to have at least six animals in the group of non-survivors. In the short-term experiments, a minimum of five animals was accepted in the group of predicted non-survivors in order to limit the number of animals suffering from a high severity of illness. Comparisons between groups were analyzed with ANOVA and Tukey’s post hoc test, as appropriate. For data in Table 1, cytokines and cardiac biomarkers were not normally distributed and were therefore presented as median, interquartile range and tested using the Kruskal-Wallis test followed by post hoc test (Dunn) correcting for multiple testing. The quality of heart rate and its changes as diagnostic tests were described by the area under the ROC (receiver operator characteristic) curve (AUC). Fisher’s exact test was used to compare positive and negative predictive values between groups. A *p* value below 0.05 was considered significant, and all hypothesis testing was two-tailed. Prism 6 (GraphPad Software, La Jolla, CA, USA) was used to perform statistics and draw the figures.

**Table 1:** Physiological and laboratory variables

Variables	Sham, baseline	Sepsis, 4 h *n* = 16		Sepsis, 24 h	*p* value (ANOVA)
	*n* = 9	Predicted survivors *n* = 11	Predicted non-survivors *n* = 5	*n* = 6	
Physiological variable					
Rectal temperature [°C]	37.4 (0.3)	38.5 (0.4)*	39.1 (1.3)*	38.1 (0.8)	*0.003*
Hemodynamic variables					
Heart rate [1/min]	346 (39)	383 (44)	444 (91)*	418 (41)	*0.012*
Change in heart rate from baseline [1/min]	not applicable	− 71 (53)	78 (24)$¶	−19 (26)	*< 0.001*
MAP [mmHg]	89 (13)	94 (20)	91 (19)	91 (15)	0.949
Arterial blood gases					
PaO_2_ [kPa]	9.63 (1.97)	9.62 (2.57)	9.21 (1.75)	8.96 (2.62)	0.946
PaCO_2_ [kPa]	5.07 (0.96)	5.50 (1.26)	5.21 (0.81)	7.12 (2.29)	0.089
pH	7.38 (0.05)	7.40 (0.03)	7.43 (0.06)	7.36 (0.11)	0.406
Base excess [mmol/L]	− 2.6 (4.7)	0.6 (5.4)	0.7 (3.0)	2.7 (2.4)	0.302
Lactate [mmol/L]	1.0 (0.3)	1.2 (0.2)¶	1.6 (0.7)	1.9 (0.8) £	*0.014*
Glucose [mmol/L]	13.0 (1.7)	12.6 (2.8)	15.6 (2.5)	11.3 (1.4)	0.055
Hematocrit [%]	34 (3)	34 (7)	45 (4)*$	37 (7)	*0.011*
Cytokines (median, IQR)					
Interleukin-1 beta [pg/mL]	72 (57–92)	129 (105–156)	332 (81–421)*¶	58 (19–91)	*0.001*
Interleukin-6 [pg/mL]	0 (0–0)	236 (138–691)*	3062 (134–8190)*	109 (0–157)	*< 0.001*
Interleukin-10 [pg/mL]	116 (59–149)	182 (155–493)	573 (114–1051)	258 (113–367)	*0.027*
TNF alpha [pg/mL]	33 (0–49)	93 (63–154)*¶	135 (47–166)	35 (0–37)	*< 0.001*
CXCL-1 [pg/mL]	195 (51–497)	877 (650–1492)*	1562 (580–1773)*	339 (111–454)	*0.001*
MCP-1 [pg/mL]	1.6 (1.3–1.6)	4.9 (3.4–5.5)*	9.3 (3.1–14.4)*	5.4 (1.1–9.1)	*0.001*
MIP-1 [pg/mL]	8 (8–14)	50 (15–150)*	262 (71–474)*¶	17 (1–21)	*< 0.001*
Cardiac biomarkers (median, IQR)					
Troponin [ng/mL]	0.0 (0.0–0.03)	0.0 (0.0–0.0)	0.0 (0.0–0.05)	0.0 (0.0–0.06)	0.152
BNP [pg/mL]	0.4 (0.2–0.8)	0.3 (0.2–0.5)	0.0 (0.0–0.3)	1.0 (0.3–2.5)	0.099

Values are shown as mean (SD), except for cytokines and cardiac biomarkers, where data were not normally distributed (median, interquartile range = IQR). Groups were compared using ANOVA (for normally distributed data) or Kruskal-Wallis test (for not-normally distributed data) followed by according post hoc tests correcting for multiple testing. Significant group differences (*p* < 0.05): *sham baseline versus sepsis 4 h, $predicted survivors versus non-survivors, ¶sepsis 4 h versus sepsis 24 h, £ sham baseline versus 24 h. *BNP* B-type natriuretic peptide; *CXCL-1* chemokine (C-X-C motif) ligand 1, a neutrophil chemoattractant involved in clearance of bacterial infections; *MAP* mean arterial pressure; *MCP-1* monocyte chemoattractant protein-1, primarily secreted by activated monocytes and macrophages; *MIP-1* macrophage inflammatory protein-1, chemotactic cytokine produced by macrophages upon endotoxin activation; *TNF* tumor necrosis factor

## Results

### Animal model

**Fig. 3:**
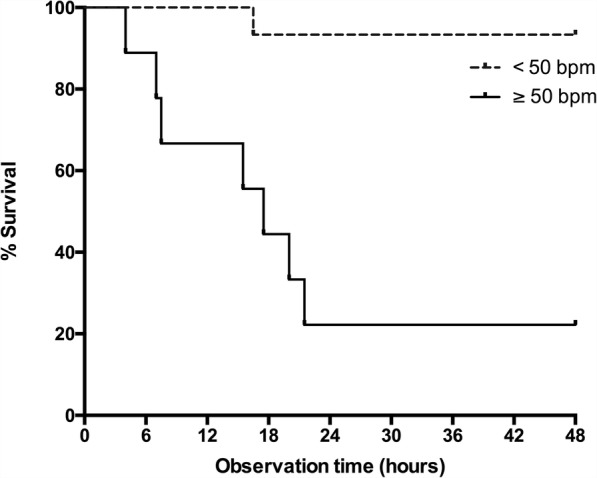
Kaplan-Meier survival curves in septic animals according to the change in heart rate between baseline and 4 h of sepsis. Twenty-four septic Wistar rats were observed for 48 h. Survival curves are shown for animals with a change in heart rate between baseline and 4 h of < 50 bpm (dashed line; mortality 1/15 = 7%) and those with a change ≥ 50 bpm (continuous line; mortality 7/9 = 78%), log rank *p* < 0.001

**Fig. 4:**
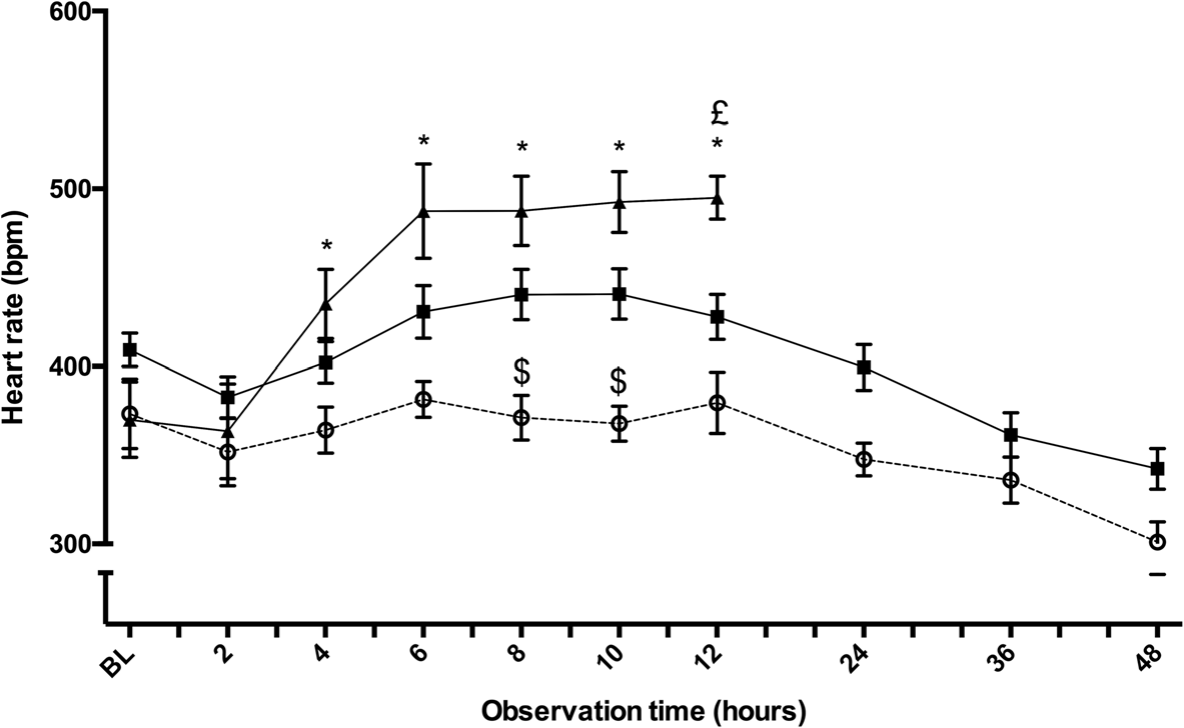
Heart rate in septic animals. Temporal changes of telemetry-monitored heart rate after induction of sepsis in 8 non-survivors (black triangles), 16 sepsis survivors (black squares), and 6 sham animals (open circles). Baseline refers to the timepoint 15 min after slurry injection in septic animals (or 15 min after end of instrumentation in sham animals). Symbols indicate means (SEM). Significant group differences (*p* < 0.05): *sham versus non-survivors, $ sham versus survivors, £ survivors versus non-survivors. Data of non-survivors were only displayed until 12 h, to avoid a selection bias due to mortality

**Fig. 5:**
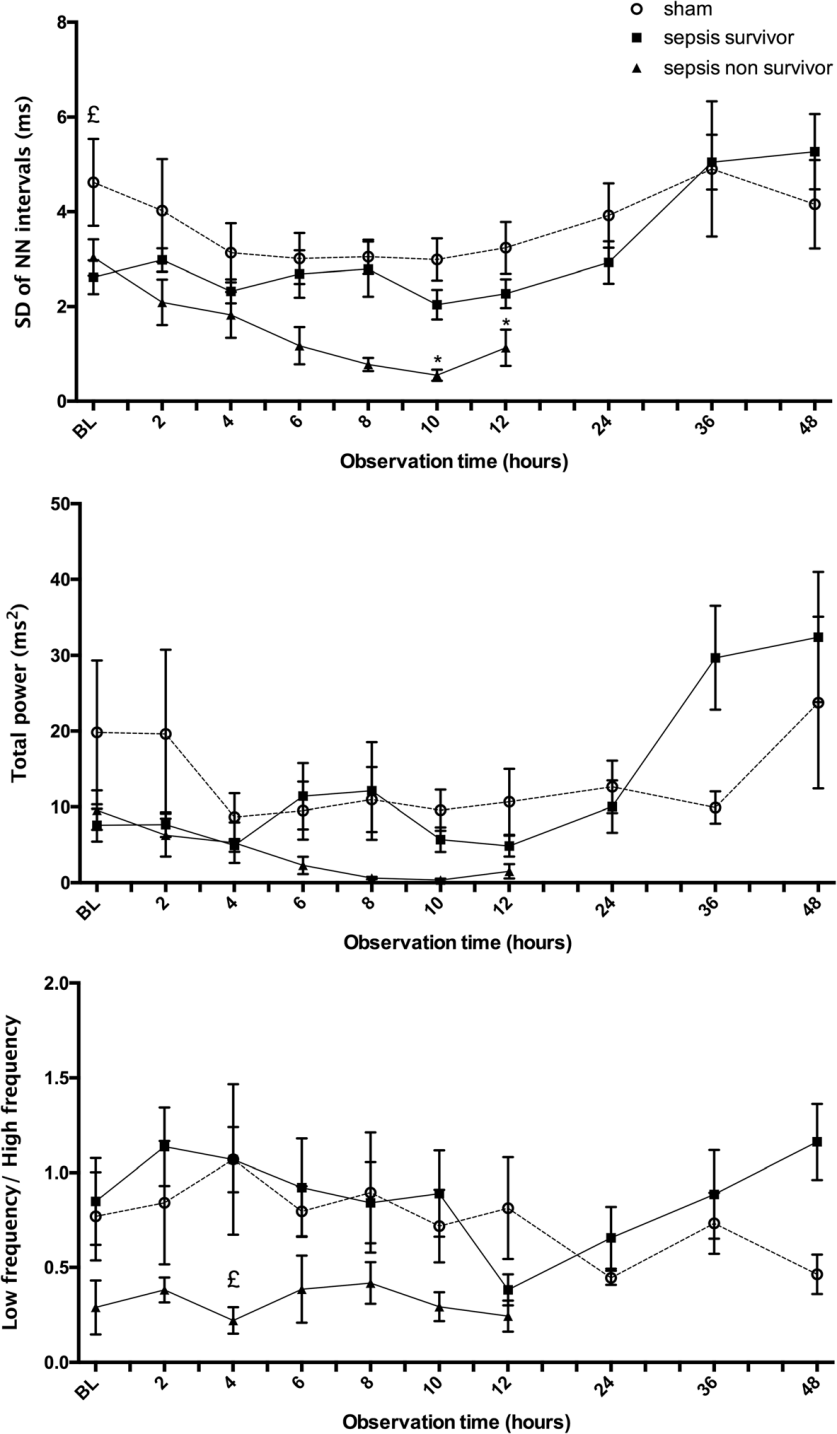
Heart rate variability in septic animals. Baseline refers to the timepoint 15 min after slurry injection in septic animals (or 15 min after end of instrumentation in sham animals). In the top figure, temporal changes of the standard deviation (SD) of normal-to-normal (NN) inter-beat intervals are depicted. Total power (TP) is shown in the middle figure. Both represent global parameters of HRV. The bottom figure shows the ratio between low frequency (LF) and high frequency (HF), reflecting the relationship between sympathetic and parasympathetic activity. Symbols indicate means (SEM). Eight non-survivors (black triangles), 16 survivors (black squares), and 6 sham animals (open circles).Significant group differences (*p* < 0.05): *sham versus non-survivors, £ survivors versus non-survivors

Twenty-four septic animals (weight 396 ± 73 g) and 6 sham animals (weight 366 ± 33 g) were observed for 48 h. Septic rats became lethargic and febrile as features of acute illness. Eight septic animals (33%) died after 13.7 ± 6.6 h, with all deaths occurring between 4 and 22 h (Fig. 3). Animals surviving beyond 24 h showed signs of recovery with increased activity and improved interest in their surroundings, but developed edema and a bloated abdomen. Heart rate changes are displayed in Fig. 4. Results of the heart rate variability analysis are given in Fig. 5.

### Laboratory findings

Microbiological cultures of ascites revealed gram-negative *(Escherichia coli*) and gram-positive (*Enterococcus sp*., alpha-hemolytic streptococci, *Corynebacterium* sp.) bacteria. Blood cultures were positive for *E. coli*, *Enterococcus faecalis*, and *Staphylococcus* sp. Antibiotic-resistance tests confirmed that the *E. coli* was sensitive to ceftriaxone.

Physiological and laboratory variables were measured in 9 sham and 22 septic animals. Results are displayed in Table 1.

### Prognosticators of outcome

Compared to baseline values, changes in heart rate at 4 and 6 h were significantly different in sepsis survivors compared to non-survivors: − 7 ± 48 bpm vs 65 ± 53 bpm (*p* = 0.003) and 21 ± 53 bpm vs 120 ± 61 bpm (*p* = 0.001), respectively. Prognosticators of death in septic animals are shown in Table 2. Importantly, an increase in heart rate ≥ 50 bpm during the first 4 h of sepsis occurred in 7 of 8 non-survivors (sensitivity 88%) and 2 of 16 survivors (specificity 88%), Fisher’s exact test *p* = 0.001. Hence, the positive and negative predictive values for this heart rate cut-off level were 78 and 93%, respectively.

**Table 2:** Prognosticators of death in septic animals

Physiological variables	ROC AUC	95% CI	*p* value
HR at 4 h (*n* = 24)	0.664	0.409–0.919	0.198
HR at 6 h (*n* = 23)	0.732	0.507–0.957	0.082
HR change between baseline and 4 h (*n* = 24)	0.875	0.684–1.00	*0.003*
HR change between baseline and 6 h (*n* = 23)	0.879	0.681–1.00	*0.005*

*AUC* area under the curve, *CI* confidence interval, *h* hours, *HR* heart rate, *ROC* receiver operator characteristic. By 6 h, one septic animal had died and could not be used for ROC calculations

Parameters from septic animals sacrificed at 4 h were tested for their prognostic potential to discriminate between predicted survivors and non-survivors (Table 3). Only hematocrit (AUC 0.93, 95% CI 0.779–1.0, *p* = 0.008) and MIP-1 (AUC 0.82, 95% CI 0.604–1.0, *p* = 0.047) were statistically different between the two groups.

**Table 3:** Parameters at 4 h and their potential to discriminate between potential survivors and non-survivors

Parameter	ROC AUC	95% CI	*p* value	Threshold	Sensitivity%	95% CI	Specificity%	95% CI
Hct (%)	0.93	0.779–1.0	*0.008*	< 43.5	100	72–100%	80	28–99%
Temperature (°C)	0.57	0.166–0.968	0.673	> 38.0	60	15–95%	83	52–98%
Lactate (mmol/L)	0.68	0.319–1.0	0.257	> 1.49	60	15–95%	100	72–100%
Base excess	0.56	0.264–0.863	0.692	< 2.20	80	28–99%	45	17–77%
pH	0.59	0.266–0.916	0.571	> 7.43	40	5–85%	82	48–98%
Glucose	0.80	0.554–1.0	0.066	> 15.5	60	15–95%	90	56–100%
IL-1a (pg/ml)	0.58	0.237–0.926	0.610	> 57.2	40	5–85%	91	59–100%
IL-1b (pg/ml)	0.56	0.194–0.933	0.692	> 244	60	15–95%	82	48–98%
IL-6 (pg/ml)	0.67	0.326–1.0	0.282	> 2775	60	15–95%	91	59–100%
IL-10 (pg/ml)	0.60	0.217–0.983	0.533	> 538	60	15–95%	91	59–100%
TNF-α (pg/ml)	0.51	0.168–0.850	0.955	< 61	40	5–85%	82	48–98%
CXCL-1 (pg/ml)	0.69	0.349–1.0	0.234	> 1885	80	28–99%	82	48–98%
MCP-1 (pg/ml)	0.56	0.211–0.916	0.692	> 7.4	60	15–95%	82	48–98%
MIP-1 (pg/ml)	0.82	0.604–1.0	*0.047*	> 211	60	15–95%	91	59–100%
BNP (pg/ml)	0.76	0.521–1.0	0.101	< 0.15	60	15–95%	82	48–98%

Animals were sacrificed at 4 h prior to fluid resuscitation and categorized according to their heart rate changes from baseline (≥ 50 bpm = predicted non-survivors; < 50 bpm = predicted survivors). This cut-off was determined from 48 h mortality experiments (see main manuscript for details). *CI* confidence interval, *ROC AUC* area under the receiver operator characteristics curve

## Discussion

We describe a clinically relevant rat model of sepsis with a 48 h mortality rate of 33%. The animals developed a typical sepsis phenotype with clinical signs of illness (reduced activity, fever), tachycardia, elevated plasma cytokine levels, positive blood cultures, and peritonitis on postmortem examination. All deaths occurred between 4 and 22 h while animals surviving beyond this range showed clear signs of clinical recovery. Heart rate changes during early sepsis were prognostic for 48 h mortality. At 4 h an increase in heart rate ≥50 bpm over baseline predicted mortality with positive and negative predictive values of 78% and 93%, respectively.

The current sepsis model is based upon a well-established rat model of fecal peritonitis [5, 8] but to which antibiotics and a continuous infusion of the potent opioid analgesic, nalbuphine [10, 11] have now been added. Injection of intra-peritoneal fecal slurry in non-antibiotic-treated animals results in polymicrobial sepsis with positive blood cultures, while post-mortem examination demonstrates generalized peritonitis and ascites formation. Injection of fecal slurry allows an identical insult in a batch of animals while avoiding a laparotomy reduces surgical trauma and postoperative pain.

Septic animals developed an early tachycardia that progressed, especially in eventual non-survivors, notwithstanding aggressive intravenous fluid resuscitation commencing from 4 h. Mechanisms such as sympathetic overstimulation are likely present, despite the use of continuous opioid analgesia to manage pain. Afferent fibers in the periphery can sense inflammation and directly activate autonomic centers within the brainstem [15]. This could be particularly important with peritonitis as the region around the celiac axis has a rich supply of sympathetic afferents [15].

The prognostic finding of tachycardia mirrors findings from patients with both non-septic [16] and septic [17–20] conditions. In our current study, an increase in heart rate ≥50 bpm during the first hours of sepsis strongly prognosticated a poor outcome. Our data support the hypothesis that outcome is determined at an early stage [21]. In our model, using an identical insult in animals of similar genotype, age, sex and upbringing, this was true even before commencement of fluid resuscitation and antibiotics. Hence, predicted non-survivors did not benefit from these standards of care, highlighting the need for new therapeutic concepts to improve survival in this group. The risk for predicted survivors is harm from additional interventions [22]; this may result in no overall benefit for the sepsis population as a whole.

Tachycardia persisted in non-survivors despite aggressive fluid resuscitation suggesting that tachycardia was not only a result of insufficient preload, but likely also a sign of autonomic dysfunction. Significant differences in HRV were found between groups, suggesting autonomic dysfunction in septic animals, particularly in the non-survivors. The ratio between low frequency (LF) and high frequency (HF) heart rate variations was significantly decreased in non-survivors. While parasympathetic tone (HF variation of heart rate) dominates over sympathetic tone (LF variation) under physiological conditions, LF/HF variability could be lost during critical illness. As described in patients with sepsis [23] and heart failure [24], autonomic regulatory dysfunction can lead to a low LF/HF ratio despite a high level of sympathetic activation. In agreement with our preclinical study, a low LF/HF ratio was associated with an increased risk of death in septic patients [25–27]. Our results are also comparable to sepsis studies in rodents [28, 29] and humans [30], in which similar results of reduced HRV have been observed. The interruption of specific feedback mechanisms between the autonomic nervous system and the heart is termed uncoupling of the components [31]; autonomic dysfunction may thus represent a potential mechanism resulting in death. Further clinical studies on heart rate and heart rate variability in septic patients are needed to better discriminate between compensatory tachycardia (adaptive) and non-compensatory tachycardia (maladaptive), and to understand when tachycardia should be treated with beta-blocking agents.

The stability of gas exchange and metabolic acidaemia at the 24 h timepoint suggest that respiratory failure or renal dysfunction is not directly responsible for mortality in this model. The absence of elevated troponin levels suggests that myocardial injury was also not a hallmark. However, elevation in BNP at this timepoint is indicative of myocardial dysfunction and mirrors clinical findings in patients with established sepsis, particularly in those with a poor prognosis [32–35]. This finding implies heart failure may be an important mechanism underlying death.

Mean cytokine levels were non-significantly higher in predicted non-survivors, albeit with high variability. Cytokine levels had normalized by 24 h in survivors, which concurrent with features of clinical recovery of signs. A similar pattern was reported by Recknagel et al. in their rat model of polymicrobial sepsis [36].

### Limitations of the study

We cannot report on circulating catecholamine levels, as this model does not allow repetitive and/or non-stressful blood sampling in awake animals. The 4 and 24 h blood samples were collected after prior surgical exposure of the carotid artery, which would invalidate catecholamine measurements at these particular timepoints.

All animals received the same fluid protocol. As shown in Table 1, predicted sepsis non-survivors had an increased hematocrit compared to sepsis survivors and sham animals, which may indicate intravascular hypovolemia. However, administration of larger amounts of intravenous fluids could be harmful for potential survivors and sham animals [5]. Fluid administration titrated to physiological parameters might offer further improvement of the model. However, heart rate alone is not useful to guide fluid resuscitation, as non-compensatory tachycardia can persist after fluid resuscitation [5].

HRV is influenced by heart rate [37] and respiration rate [38], which both changed substantially in septic rats, particularly in the non-survivors. We did not perform any intervention to directly test whether the recorded frequency bands (LF or HF) correlate with sympathetic and parasympathetic responses in our model. Furthermore, there was no recovery period between telemetry implantation and baseline measurements. While surgery and anesthesia have likely influenced heart rate and heart rate variability, this would affect all experimental groups. Potentially, the differences found in our study could be even more pronounced if there was a recovery phase between telemetry implantation and induction of peritonitis. However, a recovery phase would also increase the risk of wound infections or adverse effects of empirical antibiotic therapy that could also affect the results of the study.

## Conclusions

We describe a fluid-resuscitated rat model of abdominal sepsis with a representative sepsis phenotype and long-term mortality. This model represents not only an alternative to the standard mouse CLP model, but one more representative of human sepsis with respect to physiological, metabolic, and transcriptome changes [39, 40]. Telemetry-monitored changes in heart rate as early as 4 h after the septic insult predicted death with good sensitivity and specificity. Previous studies have demonstrated that autonomic dysfunction occurs during sepsis. The HRV analysis of this study supports this finding, but the results must be interpreted with care due to several limitations. Whether autonomic dysfunction during sepsis is a mechanism leading to death requires further mechanistic studies with a special focus on HRV. Further investigations of adaptive changes in potential survivors and mechanisms of death in potential non-survivors are possible with this model. It will also allow testing of novel treatment to assess their beneficial and harmful effects in predicted sepsis non-survivors and survivors.

## Abbreviations

AUC: Area under the curve
BNP: B-type natriuretic peptide
BPM: Beats per minute
CI: Confidence interval
CLP: Cecal ligation and puncture
ECG: Electrocardiogram
HR: Heart rate
LPS: Lipopolysaccharide
ROC: Receiver operating characteristics
SD: Standard deviation
SEM: Standard error of the mean
